# Clinical Ecopsychology: The Mental Health Impacts and Underlying Pathways of the Climate and Environmental Crisis

**DOI:** 10.3389/fpsyt.2021.675936

**Published:** 2021-05-21

**Authors:** Myriam V. Thoma, Nicolas Rohleder, Shauna L. Rohner

**Affiliations:** ^1^Psychopathology and Clinical Intervention, Institute of Psychology, University of Zürich, Zurich, Switzerland; ^2^University Research Priority Program “Dynamics of Healthy Aging,” University of Zürich, Zurich, Switzerland; ^3^Friedrich-Alexander University Erlangen-Nürnberg, Chair of Health Psychology, Erlangen, Germany

**Keywords:** mental health, mental disorder (disease), climate change, vulnerability, resilience

## Abstract

Humankind is confronted with progressing climate change, pollution, environmental degradation, and/or destruction of the air, soil, water, and ecosystems. The climate and environmental crisis is probably one of the greatest challenges in the history of humankind. It not only poses a serious current and continuing threat to physical health, but is also an existing and growing hazard to the mental health of millions of people worldwide. This synergy of literature provides a current summary of the adverse mental health impacts of the climate and environmental crisis from the perspective of Clinical Psychology. Furthermore, it presents potential underlying processes, including biological, emotional, cognitive, behavioral, and social pathways. The existing data suggest that the climate and environmental crisis not only acts as a direct stressor, but can also exert a detrimental impact on the various pathways, with the potential to amplify an individual's biopsychosocial vulnerability to develop mental ill-health. This is a call for an increased investigation into this emerging research field of Clinical Ecopsychology by clinical psychologists and other researchers.

## Introduction

The human impact on the Earth has become so meaningful that the present geological epoch has been termed the “Anthropocene”, i.e., a time of significant human-created geological change (1). This definition of the Anthropocene gives equal consideration to the potentially positive, as well as negative, imprints of humankind on Earth. On a positive note, the progressively globalized social system, a core attribute of the Anthropocene (2), has led to the unprecedented opportunity to share and grow knowledge across the globe, to promote social connections, and to allow an unparalleled global mobility. On a negative note, the rapidly growing human population has had a large-scale impact on the Earth in the form of an unprecedented pollution, environmental degradation, destruction of the air, soil, water, and ecosystems, as well as the mass destruction of species across the globe. The human imprint has triggered climate and environmental changes that have the potential to endanger the survival of the human race. In fact, the growing existential threat posed by this anthropogenic climate change has led 11,258 scientists from over 150 countries to come together to warn people about the climate emergency (3). The anthropogenic climate change is not only regarded as “…potentially the biggest global health threat in the twenty-first century” [(4), p. 1,693], but is arguably one of the most important challenges in the history of humankind.

**Figure 1:**
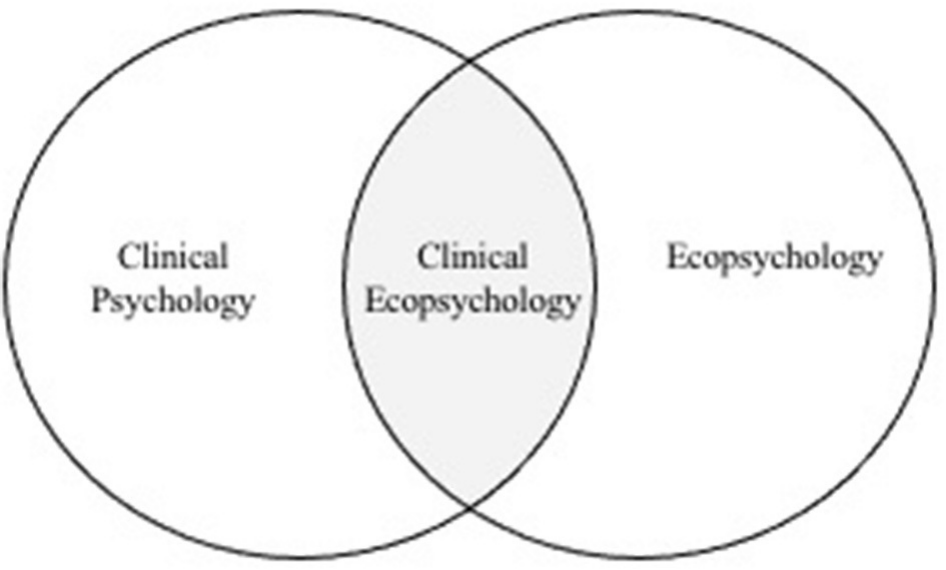
Placement of the research field “Clinical Ecopsychology” between “Clinical Psychology” and “Ecopsychology”.

This human-driven climate and environmental crisis can both directly and indirectly influence health and well-being. While the focus of prior research was directed toward physical health, comparatively little research exists on the mental health impacts. However, the investigation of the mental (ill-)health implications of the progressing climate change, pollution, environmental degradation, and/or destruction of the air, soil, water, and ecosystems is a rapidly expanding research field (5–9). The growing interest in this area can be demonstrated by the number of publications on the topic “climate change and mental health” in PubMed, which sharply increased in 2020 with 120 new publications, compared to just 69 in 2019. This research area warrants particular consideration, given the evolving urgency of the climate and environmental crisis, combined with the growing body of evidence of a substantial impact by the ongoing crisis on mental health, and paired with the exponentially growing number of publications on the topic. Special attention can be focused on evolving phenomena such as this by the provision of a distinct and precisely defined area within which to capture such research. As such, we herewith propose “Clinical Ecopsychology” as an umbrella term for existing and future research efforts that are dedicated to fostering the understanding of the development of mental ill-health in response to the climate and environmental crisis. Given that Clinical Ecopsychology may be best described as being an overlapping field of “Clinical Psychology” and “Ecopsychology” (see Figure 1), both research fields will be described in more detail in the following sections. As this work is written from the perspective of Clinical Psychology, a stronger focus is placed on this particular field.

### Clinical Psychology

Clinical Psychology is a discipline that is involved in the research, assessment, diagnosis, and treatment of mental ill-health [e.g., (10)]. At the core of Clinical Psychology lies the motivation to understand the complex nature of the etiology of mental ill-health, as well as the determination to optimally diagnose psychopathology. The overarching aim is to develop new, further improve existing, and supply efficient and evidence-based treatment options. The *Diagnostic and Statistical Manual of Mental Disorders*, in its fifth edition [DSM-5, (11)], as well as the 10th (and soon 11th) *Revision of the International Statistical Classification of Diseases and Related Health Problems* [ICD-10, (12)], assist clinical psychologists and others in the diagnosis of mental health disorders for research and practice. Clinical Psychology can thus offer the expertise and knowhow to investigate, assess, describe, and diagnose potentially psychopathological responses to the current climate and environmental crisis. To better understand the impact of the climate and environmental crisis on mental health, we can therefore draw from models in Clinical Psychology.

A central model in Clinical Psychology is the vulnerability-stress model (13, 14), according to which mental health disorders develop as a result of an interaction between the presence of stress and an individual's vulnerability (or sensitivity) to stress. This vulnerability results from the interplay of biological, psychological, and social dimensions. The biological dimension encompasses aspects related to physical health and physiological functioning, including genetic, hormonal, and immunological makeup; but also (mal)nutrition, (non-)communicable diseases, and injuries. The psychological dimension includes emotional, cognitive, and behavioral aspects, such as dysfunctional emotion regulation strategies or negative cognitive schemata. The social dimension includes aspects such as interpersonal conflicts or loneliness. Depending on the interplay of these factors, vulnerability is formed that renders an individual more or less susceptible to stress and the subsequent development of mental ill-health (13, 14).

Existing findings on the mental health implications of various climate and environmental stressors support the notion that these stressors can exert a meaningful impact on mental health *via* biological, psychological, and social pathways. Furthermore, these pathways do not act in isolation, but rather interact with each other, as can be shown in a study with Inuit in Canada (15): It was found that climate change-related alterations to the natural environment (e.g., ice instability) profoundly interfered with their traditional, land-based lifestyle, i.e., how they work, travel, and gather food (behavioral pathway). This in turn negatively affected diet and activity patterns (biological pathway), cultural identity, connection to the land, sense of place, and self-worth (cognitive pathway). This was further linked to despair, sadness, or hopelessness (emotional pathway); and can also impact social behavior (social pathway) [see also (16)]. The Inuit situation is a very good living example, which impressively shows the diversity of the impact of a changing natural environment, and the interwovenness of the underlying pathways. Through the application of the vulnerability-stress model (13, 14), this example demonstrates how Clinical Psychology can offer a useful explanatory model that can be applied in the understanding of the development of mental ill-health in response to the current climate and environmental crisis.

Current directions in Clinical Psychology include, but are not limited to, the combination of clinical psychological research with collected knowledge from clinical experience and practice, the development of evidence-based treatment guidelines, cross-cultural considerations in the diagnosis of psychopathology and culturally responsive care, the facilitation of low-threshold accesses for the treatment of mental health problems (e.g., using e-mental health interventions), the increased attention to (the treatment of) mental (ill-)health in adults of advanced age, and the adopting of a multisystem perspective on resilience [e.g., (17–19)]. These current directions in Clinical Psychology align with the demands posed by the climate and environmental crisis. It is therefore important not to miss the opportunity to apply the expertise of the large and influential field of Clinical Psychology to the current and constantly evolving climate and environmental crisis. In fact, the field of Clinical Psychology is becoming increasingly aware of the mental health impacts of the crisis [e.g., (20)]. For example, this increasing awareness was reflected in the organization of the first *International Summit on Psychology and Global Health* in 2019, held in Portugal, which was attended by leaders of multiple psychological associations from over 40 nations (21). At this gathering, the leaders declared an agreement to employ psychological research to fight climate change.

However, despite the readiness of Clinical Psychology to address the climate and environmental crisis, certain barriers exist that may also hinder an in-depth dedication to this emerging research field. Such barriers include, but are not limited to: (a) the vast complexity of the anthropogenic climate and environmental crisis, and the resulting difficulty to attribute a causal link between the climate and environmental crisis and mental ill-health (22); (b) the multiple possible direct and indirect impacts on various biopsychosocial life-domains, which are challenging to assess; (c) the general complexity of the etiology of mental health disorders; (d) the potential personal reluctance to deal with this topic due to a lack of knowledge (e.g., regarding the human-nature relationship), personal fears, or potential denial of the climate and environmental crisis; and finally, (e) clinical psychologists working in research or clinical practice may have to focus on more immediate or urgent priorities. For instance, while the Covid-19 crisis has shed light on the importance of mental health [e.g., (23)], it has also undeniably upstaged the ongoing climate and environmental crisis. Targeting such barriers can help facilitate the effective integration of Clinical Psychology in addressing the climate and environmental crisis.

### Ecopsychology

Ecopsychology has “…a healthy diversity in conceptions” [see for instance, (24), p. 2], which can make it difficult to provide an all-encompassing, commonly-accepted definition of Ecopsychology. Broadly, Ecopsychology can be described as the research field dedicated to the study of the connection between human-caused changes to and destruction of the natural world “…and the spiritual or psychological crises resulting from our increasing experience of separation from the more-than-human world. Ecopsychology looks for the roots of environmental problems in human psychology and society and for the roots of some personal and social problems in our dysfunctional relationship to the natural world.” [(25), p. 68]. “Ecopsychology explores humans' psychological interdependence with the rest of nature and the implications for identity, health and well-being. Ecopsychology topics include emotional responses to nature; the impacts of environmental issues such as natural disasters and global climate change; and the transpersonal dimensions of environmental identity and concern” [(26), Ecopsychology section, para. 1].

Ecopsychology is one of six core sub-fields of Environmental Psychology, in addition to “Behavior and the Built Environment,” “Conservation Psychology,” “Psychology on the Population Level,” “Teaching Psychology for Sustainability,” and “Using Film to Demonstrate Environment and Behavior” [(27), Interest Areas section]. According to Scull (25), in addition to its connection to Environmental Psychology, the field of Ecopsychology is related to several other larger fields, such as Psychotherapy, Spirituality, and Philosophy. Ecopsychology itself is comprised of multiple sub-disciplines, which include (but are not restricted to) sub-categories, such as Nature, Spirituality, Transpersonal Psychology, Environmental Activism, Experiential Environmental Education, Human Ecology, and Ecotherapy and Nature Connection (25). As such, Ecopsychology can be understood as a multi-faceted field comprised of numerous sub-disciplines that are all dedicated to the various aspects of the human-nature interrelationship.

The therapeutic focus of Ecopsychology has been explicitly stated (24), and is a core component of this field [e.g., (28)]. This relates to the assumption of Ecopsychology that human beings are closely attached to nature. This close emotional and spiritual bond and connection to nature is proposed to be due to the phylogenetic development of the modern human, which lasted hundreds of thousands of years and involved surviving in close interconnection with the natural world [e.g., (29, 30)]. Given this evolutionary “up-bringing” in nature, living in equilibrium with nature is thought to be a crucial prerequisite for humans' physical and mental health and well-being (31). While Ecopsychology and Clinical Psychology both have their place in understanding the mental health impacts of the climate and environmental crisis, a distinct research area drawing on the most relevant elements of each field can help advance this research topic further.

### Clinical Ecopsychology

Clinical Ecopsychology seeks to systematically examine the direct and indirect mental health impacts of the progressing climate change, pollution, environmental degradation, and/or destruction of the air, soil, water, and ecosystems. More specifically, Clinical Ecopsychology sets out to examine underlying pathways of how the climate and environmental crisis can lead to the development of mental ill-health, which entails the study of specific risk and vulnerability factors. Clinical Ecopsychology also investigates potential protective and resilience factors that may aid individuals in their adaptation and that may foster the maintenance of mental health in the face of the ongoing crisis. Clinical Ecopsychology further aims to thoroughly examine the beneficial mental-health impact of contact with the natural world. Taken together, Clinical Ecopsychology sets out to examine underlying pathways that lead to the development of mental ill-health in the face of ecological adversity. This involves the study of risk, vulnerability, and protective factors to understand interindividual differences in the response and adaptation to ecological adversity, and the promotion of (the creation of) efficient treatment options for mental health disorders linked to the climate and environmental crisis.

It must be noted that Clinical Ecopsychology is not a new research field. Both Clinical Psychology, but in particular Ecopsychology (in addition to Environmental and Conservation Psychology), have previously made extremely valuable contributions to the understanding of these topics [see for instance (32–37)]. As such, Clinical Ecopsychology may also be described as a respective sub-category of these fields: Clinical Ecopsychology is a sub-field of Clinical Psychology in its focus on climate and environmental stressors and its consideration of the beneficial impact of nature/natural stimuli, which is an existing, although still vastly neglected and marginal topic within Clinical Psychology. Clinical Ecopsychology is equally a sub-field of Ecopsychology, as it shares many, though not all, interests of Ecopsychology. For instance, Clinical Ecopsychology may not (or only to a very limited extent) deal with topics such as Nature Spirituality, Transpersonal Psychology, or Environmental Activism, which are sub-disciplines of Ecopsychology (25). Clinical Ecopsychology may also draw on more “clinical” language than Ecopsychology, such as when describing therapeutic processes and outcomes (e.g., to “treat” rather than to “heal”). However, while Clinical Ecopsychology may be most optimally placed between Clinical Psychology and Ecopsychology (i.e., through their shared overlap), this should not mask the fact that Clinical Ecopsychology is in itself a multi-disciplinary research field. In fact, an impressive and rich variety of disciplines (e.g., Psychiatry, Psychology, Medical Sciences, Sociology, Public Health, and Biology) have already contributed to the increasing understanding of the mental health impacts of the climate and environmental crisis. The multiple disciplines from which it is possible to examine the mental-health impact of the climate and environmental crisis mirrors the multi-dimensionality of (the development of) mental ill-health.

As stated above, Clinical Ecopsychology not only focuses on the development of mental ill-health in response to the climate and environmental crisis; but also aims to foster a better understanding of how mental health can be upheld in the face of the ongoing crisis, and how mental health disorders linked to ecological adversity can be most optimally treated. Potentially useful therapeutic approaches include the validation and management of emotional responses, verbalization of (complex) emotions, fostering active hope, finding a balance between pessimism and optimism, stimulating self-efficacy, promoting social connectedness, and training adaptive emotion regulation strategies [e.g., (38)]. Cognitive strategies can also be used to foster functional cognitive coping mechanisms, such as cognitive reframing, problem solving, reflection of responsibility and own contribution, putting negative thoughts into perspective, and radical acceptance [e.g., (39, 40)]. Depending on the needs of the individual, single or group settings should be offered, and low-threshold options should be made available, such as informal meetings with like-minded individuals. These informal meetings may be particularly important, as previous research has shown that becoming active through collective group engagement is linked to hope, a key protective factor in dealing with eco-anxiety: “Hope can help them confront the problem and bear the burden of taking on climate change without becoming overwhelmed” [(41), p. 13]. Furthermore, given the positive impact of natural stimuli, contact with nature and natural elements should be integrated whenever possible into psychotherapeutic work on the topics of Clinical Ecopsychology [for an overview see (42)]. For instance, research on a form of nature therapy called Shinrin-yoku, in which individuals spend time in the forest, has shown reductions in mental fatigue through decreased prefrontal cortex activity, reduced cortisol concentrations, and increased feelings of comfort and calm (43). The deep-rooted psychophysiological connection to nature may help individuals to (sub-)consciously resonate with the natural environment and to respond positively to its restorative influence (44–47).

In conclusion, it is the aim of Clinical Ecopsychology to combine existing multi-disciplinary forces in order to create, share, and promote the necessary knowledge required for: (a) raising awareness of the increasing psychopathology stemming from anxiety, frustration, and despair associated with the climate and environmental crisis; (b) the maintenance of mental health in the face of ecological adversity; (c) the efficient treatment of mental health disorders linked to ecological adversity; (d) increasing access to mental health care across the globe, particularly in those regions most affected by the crisis; (e) the planning and implementation of intervention measures; and (f) the provision of additional research-based arguments for the urgently needed global action on the current and worsening climate and environmental crisis.

### Aim and Structure of This Synergy of Literature

Given the wide-ranging, and diverse multidisciplinary literature on the mental health impact of the climate and environmental crisis, a systematic literature search was not considered feasible nor the goal of this report. Instead, this synergy of literature aims to extend existing research by providing a current overview of the core and most recent lines of research by summarizing the existing literature from the perspective of Clinical Psychology. The online databases PsychINFO, Web of Science, PubMed, and Scopus were used for the literature search. Furthermore, reference lists of appropriate articles were also screened for additional studies. Search terms related to the following domains were applied: mental health (e.g., mental health disorders, psychopathology), climate change (e.g., climate crisis, global warming), and environmental crisis (e.g., destruction of nature, pollution). Only peer-reviewed articles written in English and published by the end of September 2020 were considered for the synergy of literature.

This article is structured as follows: climate and environmental stressors are first described, followed by an in-depth examination of the mental health impacts of the various ecological stressors. Potential underlying processes are then summarized, encompassing biological and social pathways, as well as the previously neglected emotional, cognitive, and behavioral pathways. The way in which the climate and environmental crisis can amplify an individual's biopsychosocial vulnerability is then outlined. This is followed by a description of risk and vulnerability factors, including vulnerable populations, and the potential for increased adaptation to ecological stressors. This synergy of literature concludes with a discussion of methodological considerations and future directions.

## Climate and Environmental Stressors

The climate and environmental stressors outlined in this report refer to human-created ecological changes. “Human activities are estimated to have caused ~1.0°C of global warming above pre-industrial levels, with a *likely* range of 0.8–1.2°C. Global warming is *likely* to reach 1.5°C between 2030 and 2052 if it continues to increase at the current rate” [(48), p. 4]. The main driving force behind the increasing temperatures is greenhouse gas emissions, which are linked to economic growth, the steadily growing human population, and the excessive consumption of the wealthy lifestyle (3). Despite continuous warnings about the state of climate change since the First World Climate Conference in 1979, there is no evidence of a decrease in greenhouse gas emissions (3). The consequences of this climate change are manifold, including the loss of ecosystems and biodiversity, the increase in ocean heat and acidity, the melting of the glaciers, the rising sea level, as well as an increase in extreme weather events and natural disasters (48).

In addition to climate change, humans are also responsible for pollution of the air, soil, and water, as well as for the destruction of biospheres and ecosystems, deforestation, and resource extraction. Air pollution, largely caused by the burning of fossil fuels (49), is a major concern in urban areas, particularly in megacities and developing countries: “…91% of the world's population lives in places where air quality levels exceed WHO limits” [(50), Ambient Air Pollution section, para. 2]. Another major form of pollution comes from mismanaged plastic waste, which is detrimental for oceans and marine wildlife. It was estimated that in 2010 alone, 4.8–12.7 million metric tons of plastic waste entered the ocean (51). Pollution can also be caused by oil spills, such as the Deepwater Horizon Oil Spill in the Gulf of Mexico in 2010, with an estimated discharge of around five million barrels of oil and natural gas. This oil spill spoiled sediment, seawater, marine flora and fauna, as well as commercial seafood species, such as shrimp (52). The unprecedented decline in nature in the history of human beings (53) is so substantial that scientists describe the current loss of biodiversity as the Earth's sixth mass extinction (54). Across the globe, scientists increasingly emphasize biodiversity issues, such as the impact of the extinction of insect populations (55), the need for conservation of subterranean ecosystems (56), or the freshwater biodiversity crisis (57). Of particular concern is the trend in deforestation, with a 49.6% increase per decade in global tree cover loss over the last 20 years (3).

The effect of this climate and environmental crisis can vary depending on whether the phenomena are *acute, sub-acute*, or *chronic*, and whether the effect is experienced more *directly* or *indirectly*. Acute events refer to fast-onset disasters, such as extreme weather events or an oil spill. Sub-acute events refers to slow-onset or slow-moving disasters, such as droughts or air pollution. Chronic events refer to more subtle occurrences and insidious changes, such as the rise of sea levels or the decreased livability of habitats in certain regions (5, 9). Individuals can be directly affected by disasters *via* personal exposure, such as through injury, physiological harm, traumatization, but also by abrupt community losses (e.g., by wildfire). In many cases, individuals are indirectly affected through the various impacts of the climate and environmental crisis on their biopsychosocial life-domains. This includes indirect psychosocial impacts triggered by changes to one's socio-environment, such as by damages to the infrastructure of one's hometown by a hurricane, or by experiencing economic hardship and potentially forced migration caused by a climate-change related drought. Another indirect impact relates to the experiencing entity, i.e., the subjective, psychological impact of the climate and environmental crisis. Strong emotional reactions such as fear, sadness, hopelessness, or guilt can arise due to witnessing the crisis from a distance, such as through learning or increased virtual exposure, i.e., confrontation with media representations about the climate and environmental crisis (5, 38, 58, 59). All of these described direct and indirect impacts can co-exist, overlap, and be nested within each other.

While accumulated research evidence has demonstrated physical health effects [e.g., (60)], the investigation of the mental health impacts of climate and environmental stressors is a comparatively recently emerging research area, which can be considered within the frame of Clinical Ecopsychology.

## Impact on Mental Health

This section provides a current overview of studies investigating the mental health impact of a variety of climate and environmental stressors.

### Impact of Extreme Weather Events and Natural Disasters

Extreme weather events (e.g., floods), and natural disasters (e.g., wildfires) have been previously linked to a broad range of adverse mental health outcomes (22, 61–66). The most commonly reported mental health disorders in the aftermath of extreme weather events and natural disasters are posttraumatic stress disorder (PTSD) and depression, as well as anxiety, suicide, and substance abuse disorders (22, 67, 68).

Large differences in prevalence rates are found in studies on the impact of extreme weather events and natural disasters on mental health [i.e., PTSD, 0.7–52.6%, anxiety disorders, −0.4 to 84%, and depression, 0.9–81%, (65)]. This may be due to multiple factors, such as differences in socio-demographics (e.g., age, sex), disasters (e.g., type, severity), magnitude of exposure, or applied methodological differences (e.g., study design, psychometric instruments) (65). In particular, assessment time plays a critical role, as psychopathological burden varies as a function of time (8). Burden appears to be higher in the short-term, reaching the highest levels within the 1st year post-disaster and attenuates thereafter, with variations in peak and trajectory depending on severity of symptomatology (22, 69). Nevertheless, despite these prevalence differences, PTSD is repeatedly observed to be the most frequently examined mental health disorder following extreme weather events and natural disasters (70). Given the particular characteristics of these events, such as the suddenness, the destructive potential, and likelihood for severe sequelae (e.g., injury, death); it is unsurprising that such ecological stressors can trigger the development of stress-related mental health disorders.

There is a lack of longitudinal studies on PTSD following extreme weather events and natural disasters, with some notable exceptions. For instance, McLaughlin et al. (62) examined the impact of Hurricane Katrina on PTSD over time. From the affected individuals, 17.1% were estimated to have PTSD at 7–19 months (baseline) and 29.2% at 24–27 months (follow-up) post-disaster. Around 40% with PTSD at baseline had recovered by the follow-up. More than 40% with PTSD at follow-up showed a delayed onset, i.e., onset later than 6 months after the hurricane (62). It should be noted that this study did not obtain pre-disaster data on psychological functioning, which prevented statements about the change in mental health in response to the disaster. However, a set of studies (63, 64, 66) was able to longitudinally compare psychopathological burden from pre- to post-Hurricane Katrina in a high-risk sample of low-income parents. At around 11 months post-disaster, Rhodes et al. (66) found that serious mental illnesses were twice as high post-disaster (13.8%) compared to pre-disaster (6.9%), with almost half the sample (47.7%) showing probable PTSD. Follow-ups were conducted with the same sample at 5 years (63), and 12 years after the hurricane (64). Probable PTSD declined from 33 to 16.7%, suggesting a slow recovery over time. Similarly, non-specific psychological distress also showed an elevation from baseline (24.0%) to the first follow-up (35.2%), with a slow decline thereafter (second follow-up: 30.1%; third follow-up: 28.5%), but remaining above baseline values (64). These studies show that more than a decade after an “acute” natural disaster, adverse mental health effects can still be detected in some survivors.

### Impact of Increasing Temperatures and Extreme Heat Event

Increasing temperatures are resulting in a higher frequency of extreme heat events, such as heat waves (48). The occurrence of these extreme heat events is a major public health problem as they have been linked to a vast array of mental health consequences, including aggressive and criminal behavior, wake and sleep disorders (e.g., insomnia, obstructive sleep apnea), depression, and suicide (71–73). For example, Burke et al. (74) observed an increase in suicide rates in the US and Mexico (0.7 and 2.1%, respectively) between 1990 and 2010, in response to a 1°C increase in average monthly temperature (74). In addition, a recent study provided large-scale evidence for the climate change—mental ill-health relationship, by combining meteorological data (between 2002 and 2012) with mental health data from almost two million US residents (75). Results showed that worse mental health was related to short-term exposure to extreme weather events, multiyear warming, and exposure to tropical cyclones (75).

Increasing temperatures have also been linked to higher admission rates to emergency departments and psychiatric hospitals in individuals with pre-existing mental health conditions. For example, in the study by Shiloh et al. (73), a significant association was found between the maximum environmental temperature and psychiatric hospital admission rates of patients with an acute psychotic exacerbation of schizophrenia. This suggests that psychotic symptomatology, such as hallucinations or delusions, can vary as a function of (higher persistent) temperatures, with disturbed sleep as a result of the higher temperatures potentially playing a role (73). These studies show the adverse impact that “chronic” events (i.e., rising temperature) can have on mental health.

### Impact of Droughts

Related to the increasing temperatures, climate change can also increase the likelihood of droughts and precipitation deficits in some world regions (48). Droughts have previously been linked to an increase in general mental distress. For instance, a study examined the mental health impact of a long-lasting drought in Australia and found that in response to the 7-year drought, distress increased in rural (but not urban) habitants by 6.22% (76). Another Australian study including 8,000 individuals found “…that if the population in rural and regional areas of Australia were not exposed to drought the incidence of mental health problems in these areas would be 10.5% lower” [(77), p. 187]. An increase in suicide rates during drought periods has repeatedly been reported, but with inconsistent findings across studies [e.g., (71)]. This research demonstrates the adverse effects of “sub-acute” events on mental health.

### Impact of Water and Food Insecurity

Rising temperatures that cause the retreat of glaciers, along with climate-change related variations in precipitation patterns, are leading to a reduction in fresh water resources and in agricultural yield. These changes affect the water and food security of millions of people worldwide, particularly those heavily dependent on rivers and precipitation (78). The impact of climate change on water and food supplies is complex and co-influenced by political, social, and environmental factors (79). Two-thirds of the world population are confronted with severe water scarcity for a minimum of 1 month per year (80); and up to 800 million people are affected by food insecurity (81). These high numbers show that water and food insecurity are large-scale risk factors for health and well-being [e.g., (82)].

Water scarcity has been linked to psychological distress, suicidality, anxiety, hopelessness, and depression [for a review see (83)]. Insecure or scarce access to water can induce worries about material deprivation, shame and stigma for failing social roles or norms (e.g., begging for water, unable to clean oneself), as well as persistent concerns about threats to physical health, such as contaminated water (83). Similarly, food insecurity, and the resulting malnutrition, has not only been linked to physical health conditions (e.g., anemia, adverse physical development), but also mental ill-health (81). A recent systematic review and meta-analysis on food insecurity and mental health found an increased risk for depression and stress (84). The association between food insecurity and depression followed a dose-response pattern, with more food insecurity associated with a higher risk for depression, with a higher risk observed for men and older adults (above 65 years) (84). These studies illustrate the negative impact that “sub-acute” events (i.e., water and food insecurity) can have on mental health.

### Impact of Air Pollution

Several studies have examined the impact of air pollution on mental health [for an overview see (85)]. Exposure to (ultra)fine particulate matter has been linked to depressive symptomatology in both cross-sectional (86) and longitudinal (87) research. In the exploratory longitudinal study by Roberts et al. (87), results showed that exposure to concentrations of air pollution at 12 years of age was associated with the development of depressive symptoms and a higher risk of being diagnosed with depression by the age of 18 (87). A recent systematic review and meta-analysis also revealed that ambient particulate matter (with an aerodynamic diameter smaller than 2.5 μm) was strongly associated with an elevated risk of depression and marginally related to a higher risk of suicide (88). Findings further suggest an accumulative detrimental impact over time, with longer exposure to particulate matter associated with greater risk of depression (88). Furthermore, studies on air pollution and suicide have shown increases in the rate of suicides of 1–2% per day of poor-quality air (71).

In addition, exposure to air pollution has also been linked to cognitive impairment, as well as to diseases affecting the central nervous system (CNS), such as Alzheimer's disease (88–90). For example, a population-based cohort study in Canada found that individuals living in proximity to major roadways with high levels of air and noise pollution had a higher risk of developing dementia (91). Up to 11% of the dementia incidence within the 11-year observation period could be attributed to the heavy traffic exposure and the related air pollution (91). These studies show the adverse impact that air pollution, a “sub-acute” event, can have on psychological and cognitive health and well-being.

### Impact of Profound Changes to the Natural Environment

Profound changes to the natural environment and landscape, either by human-created destruction (e.g., open mining), destruction through natural forces (e.g., wildfire), or as a consequence of climate change (e.g., melting glaciers), can evoke grief, sadness, and feelings of loss in (place-based) solace. These emotions have previously been examined under the terms ecological grief (16) and solastalgia (92). Ecological grief is defined as “…the grief felt in relation to experienced or anticipated ecological losses, including the loss of species, ecosystems, and meaningful landscapes due to acute or chronic environmental change” [(16), p. 275]. Similarly, solastalgia refers to “…the pain or distress caused by the loss of, or inability to derive solace connected to the negatively perceived state of one's home environment” [(92), p. 96]. Ecological grief and solastalgia have been observed in various populations across the world, such as in the form of undermined notions of place-based, cultural, and self-identity; cumulative and chronic place-based distress; amplified mental health stressors; and heightened perceived risk of depression and suicide [e.g., (15, 93)]. In particular, individuals who live in close connection with nature, such as Indigenous people or farmers, have been found to be more vulnerable to the development of ecological grief and solastalgia (16, 92, 94). These studies highlight the adverse mental health impact of “chronic” ecological events in the form of profound environmental changes.

### Impact of Increased Awareness

A recent survey of over 27,000 participants from 26 countries revealed that climate change was perceived as a major international threat for half of the participating nations (95). This indicates an increase in the perception of the threat posed by climate change: while a median of 56% reported climate change to be a top threat in 2013, this number increased to 63% in 2017, and 67% in 2018 (95). This perceived threat illustrates an indirect effect of climate change on mental health. For instance, a qualitative study with young urban children (10–12 years old) found that 82% reported feelings of sadness, fear, and anger in relation to environmental issues and 72% expressed pessimistic, negative, or apocalyptic views about the future (96). Furthermore, a poll conducted with 2,107 U.S. adults on the topic of climate change found that 56% identified climate change to be the most important problem of today's society, with 68% reporting having experienced at least a little eco-anxiety (97). Younger participants (18–34 years) were more affected, with almost half reporting climate change-related stress to have an impact on their daily lives (97).

Thus, far, vast differences exist in the definition of eco-anxiety, or climate (change) anxiety [e.g., (39, 98–100)]. This may be due to the fact that eco-anxiety can be understood quite differently from the varied perspectives of the respective disciplines (e.g., Sociology, Philosophy, or Depth-Psychology). Nowadays, some describe it as “…a specific form of anxiety relating to stress or distress caused by environmental changes and our knowledge of them” [(40), p. 1,233], while others use it to describe a range of negative climate emotions [e.g., (94)]. From the perspective of Clinical Psychology, eco-anxiety is understood as a syndrome, i.e., a set of psychological symptoms related to the broader spectrum of anxiety or fear that are triggered by (thoughts about) the climate and environmental crisis, which can cause subjective suffering and lead to functional impairments. The APA defines eco-anxiety as “…any anxiety or worry about climate change and its effects” [(97), para. 6]. From a clinical perspective, a defined set of criteria/symptoms for eco-/climate (change) anxiety do not yet exist. Instead, various symptoms have been linked to this particular anxiety, such as fear, distress, worry, sadness, or rumination (98). Currently, climate change anxiety can be assessed with a recently developed and validated measure by Clayton and Karazsia (99). It must be noted that responding to this unprecedented climate and environmental crisis with intense negative emotions is not a pathological reaction, but rather can be regarded as a normative, adequate, and justifiable response to a real existential threat [e.g., (16)]. However, if the eco-/climate (change) anxiety becomes maladaptive and so intense that it significantly impacts psychosocial functioning (i.e., interferes with everyday life functioning, causes profound suffering, or is perceived as uncontrollable), ecological syndromes such as eco-anxiety may become a clinically relevant concern warranting clinical attention (98).

Regarding this clinically relevant concern of the climate and environmental crisis, it is correct to state that the current diagnostic manuals, i.e., the DSM-5 (11), and the ICD-10 (12), do not include disorders such as eco-anxiety, climate-change phobia, prolonged eco-guilt disorder, or climate-change depression. However, it is a common misconception that is not possible to diagnose mental health disorders triggered by the climate and environmental crisis. In fact, the stressors associated with this crisis can theoretically cause a multitude of different mental health disorders, depending on the individual biopsychosocial-vulnerability constellation of the affected person (13, 14). For instance, having constant worries about the state of the climate, the continuing deforestation of rainforests, the bee mortality, or the future perspectives of one's offspring, could provide the basis for the development of a *Generalized Anxiety Disorder* (DSM-5: 300.02; ICD-10: F41.1), if the symptomatology were to cause meaningful distress or significantly impact the psychosocial functioning. Furthermore, *Adjustment Disorder* (DSM-5: 309.9; ICD-10: F43.20) is defined as “…a maladaptive reaction to an identifiable psychosocial stressor or multiple stressors that usually emerges within a month after the onset of the stressor” [(101), p. S3]. This is a suitable and useful mental health diagnosis that can be used to describe a symptomatology of distress triggered by climate and environmental stressors. However, even if individuals did not meet the diagnostic criteria for an existing mental health disorder, the suffering caused by a sub-clinical/threshold symptomatology can also diminish quality of life and may warrant clinical attention.

### Impact of a Lack of Greenness and Contact With Nature

Further evidence of the indirect effects of climate change on mental health can be seen in the rapidly ongoing trend toward urbanization, as more and more people lose contact with the natural world. The current discrepancy between the modern (urbanized) lifestyle and our psychophysiological development is thought to impact mental health. For example, the increased amygdala activity observed in city dwellers, compared to country or small-town dwellers, may be an indicator of the higher stress of city living (102). Furthermore, higher levels of urbanization have also been linked to increased psychopathology, such as psychosis and depression [e.g., (103)], and higher suicide rates (104). In addition, a recent large-scale cohort study investigated the relationship between environmental greenness and schizophrenia, using data from almost 870,000 individuals over a 10 years period (105). Results found that higher environmental greenness was associated with a lower schizophrenia incidence, suggesting that a lack of greenness may be one factor explaining why urban areas are linked to lower mental health (105).

## Underlying Processes

Climate and environmental stressors can exert an impact on mental health through multiple pathways, five of which are presented in the following section:

### Biological Pathway

In the following two sub-sections, the various physical health impacts are presented, followed by an in-depth description of psycho-pathophysiological processes induced by environmental toxins.

#### Impact on Physical Health

Given the intertwined relationship between physical and mental health [e.g., (106)], bodily harm by injuries or exposure to environmental toxins, as well as (infectious) illnesses, diseases, or physiological health conditions caused by climate and environmental stressors can increase the vulnerability for the development of mental ill-health. The impact of climate change on injury is not well-examined (107). However, existing data supports the notion that there is a link between changes in climate and the occurrence of injuries. Extreme weather events and natural disasters can increase the risk of injury. For instance, a systematic review on the impact of extreme weather events in developing countries reported an increase in injuries of 0.3–37.3% (65). Additionally, anomalously warmer temperatures, such heat waves, have been linked to an increase in deaths from unintentional injuries (e.g., falls) and intentional injuries [e.g., suicide, assault; (108)]. Heat stress was also significantly associated with an increased risk for work-related injuries, such as falls, stab-cut injuries, or road traffic injuries (109).

Heat waves have also been linked to a vast array of climate-sensitive health outcomes, including communicable and non-communicable diseases [e.g., (110, 111)]. The human body tries to adapt to heat in a number of ways, such as by down-regulating thyroid hormones, up-regulating blood levels of noradrenaline and prolactin, and increasing heart rate [e.g., (112, 113)]. Such heat stress can cause heat strain, hyperthermia, dehydration, heat-related exhaustion, heat syncope, heat cramps, and heatstroke [e.g., (112, 114)]. While healthy individuals can be affected by heat-related illnesses [for a review see (115)]; being of higher age, taking certain medications, having a chronic disease (e.g., cardiovascular diseases, obesity, and diabetes), and having mental and neurological disorders (e.g., schizophrenia) are known risk conditions for heat-related illnesses (116–118).

Air pollution, which is exacerbated by warmer temperatures, is also a risk factor for a range of chronic diseases and medical conditions. Globally, air pollution has been shown to contribute to 29% of lung cancer deaths, 24% of stroke deaths, 25% of heart disease deaths, and 43% of lung disease deaths (50). While the impact of air pollutants has been extensively studied with respect to respiratory and cardiovascular diseases, it has also been more recently examined in connection to CNS related disorders, such as Alzheimer's and Parkinson's diseases (119).

Extreme weather events, such as heavy rainfalls and floods, can give rise to water-borne diseases, which are meaningful contributors to the global burden of disease and mortality (120). Examples of water-borne or water-related diseases include gastrointestinal diseases (e.g., diarrhea, cholera), typhoid fever, viral hepatitis, or schistosomiasis (82, 120, 121). Such diseases are caused and fostered by multiple factors, including contaminated water, limited access to sanitation facilities, lack of knowledge about hygiene practices, shortage of electricity or plumbing, overcrowding, or malnutrition (79, 120, 121). These diseases are a particular concern in developing countries, which, among other risk factors that limit adaptive capability (e.g., poverty), have a higher likelihood to be severely affected by extreme weather events (121).

Vector-borne diseases are illnesses transmitted through the bite of vectors (e.g., mosquitos, ticks), whose presence and abundance are climate sensitive (122, 123). These diseases include West Nile Fever, Lyme borreliosis, tick-borne encephalitis, lupus, malaria, and dengue fever; and they have been linked to climate change-related alterations in regional and local climatic and environmental conditions (79, 122–124). The increase in vector-borne (and zoonotic) diseases is a consequences of several (interacting) mechanisms, including changes in the geographical distribution range and the population density of hosts and vectors, and changes in infection prevalence and pathogen load (development, reproduction, and replication) (124).

#### Pathophysiological Processes Induced by Environmental Toxins

Environmental toxins in the form of air pollution can be translated into psychopathology *via* biological pathways, due to their toxic effect on the CNS (85, 89). In this pathway, (long-term) exposure to environmental toxicants in the air [e.g., (ultra)fine particulate matter, nitrogen oxides, and heavy metals] has been linked to environmentally-induced oxidative stress, neuroinflammation, damage to the cerebrovascular system, and neurodegenerative pathology (88, 89, 91). Previous studies have shown that components of air pollution particles can reach the brain *via* different routes. For example, particulate matter and absorbed and soluble compounds can enter the brain directly, with consequences including neuroinflammation, microglial activation, and blood-brain barrier damage or dysfunction (89). Furthermore, a more indirect route is peripheral inflammation, which is disinhibited as a consequence of the peripheral presence of pollutants. This can exert further negative effects on the brain by circulating inflammatory cytokines (125). Together, these processes stimulate neuropathology and diseases of the CNS.

In addition, the disinhibition of peripheral inflammation can have negative long-term effects on somatic health. Inflammatory mechanisms are directly, and often causally, involved in many pathophysiological processes, such as atherosclerosis, insulin resistance, and the development of malignancies (126). Through these mechanisms, inflammation contributes to life-threatening diseases, such as cardiovascular disease, type 2 diabetes, some types of cancer, and sarcopenia [e.g., (127, 128)]; Inflammation can also have adverse mental health effects, including the development of depressive symptoms, fatigue, and long-term cognitive decline (125, 129).

### Emotional Pathway

The (topic of) climate change, pollution, and the destruction of the natural environment can impact mental health by evoking intensely negative emotions (98). This potential to induce negative emotional responses is detailed in the following sections, followed by a description of an underlying process through which triggered emotional responses can detrimentally impact mental health.

#### An Emotional Topic

The climate and environmental crisis can be understood as a severe, and rather unique current (global) stressor, which is associated with particular aspects that evoke or intensify negative emotional responses. The fact that the existence of humankind may be threatened by the consequences of this crisis links it to themes such as existential threat, destruction, or death [e.g., (39)]. These morbid associations can induce mortality awareness, which “…creates an existential terror” that can engage psychological defenses and provoke profoundly distressing emotional reactions [(130), p. 3]. The global scale of the crisis can further give rise to feelings of apathy, numbness, loss of control, powerlessness, uncertainties about the future, as well as a state of “eco-paralysis,” particularly in young people (41, 94). These feelings can be further exacerbated by observing the apparent inaction by global leaders and the general public to mitigate or adequately address the crisis. In addition, the impact of the climate and environmental crisis is unequally distributed across the globe, often most affecting those who contributed least (131, 132). This issue of injustice is a component of the crisis that has the potential to induce strong emotional reactions, including anger, and (ecological) guilt (133).

Furthermore, human-created/technological disasters and interpersonal traumatic experiences have commonly been linked to worse mental health outcomes in comparison to natural disasters, such as volcanic eruptions [e.g., (70)]. However, extreme weather events are more common and intense due to the magnitude of the anthropogenic climate change. This adds a human-created component to these events and blurs the division between natural and human-created disasters (58). The growing awareness of the anthropogenic aspect to climate change and related consequences, as well as the obvious human-created elements of pollution and the destruction of the environment, may have negative effects on mental health (59).

Lastly, the natural world bears an emotional importance for human beings (45). According to Wilson's *Biophilia Hypothesis* (134), humans have an inherent emotional affiliation to the natural world, and “…an innate tendency to focus on life and lifelike processes” [(30), p. 1]. Individuals living in close connection to the natural world can experience this bond to nature as similar to that with a person. For example, in interview research with Inuit in Canada, one participant reported that “…nature's about, to us is like in a way, another person” [(135), p. 19]. Observing the pollution and destruction of the natural world can thus psychologically hurt individuals, induce feelings of distress, and trigger emotional misery, particularly in those with close bonds to nature.

#### From Negative Emotions and Distress to Ill-Health

Longer-term experiences of stress, anxiety, or depression are associated with altered basal activity of the main stress systems: the sympathetic nervous system (SNS) and the hypothalamus-pituitary-adrenal axis (HPA axis). The SNS tends to be over-activated in individuals experiencing chronic stress, anxiety, or depression, which is reflected by higher plasma concentrations of epinephrine and norepinephrine, and is typically associated with increased blood pressure, heart rate, and other sympathetically controlled functions (136). The HPA axis mainly shows alterations in its diurnal rhythm in response to stress, with the specific changes depending on the duration and type of stressor. For instance, for chronic stressors with a duration of several months, the majority of studies report a flattened diurnal activity rhythm and markedly lower plasma cortisol concentrations, a state called hypocortisolism (137, 138). Alterations in these two stress systems converge in their effect on dependent systems to become a threat to health. One important dependent system is the inflammatory system, which can be regulated by stress systems, with the SNS typically exerting a stimulating effect on inflammatory mechanisms and the HPA axis exerting anti-inflammatory effects [for example see (139)]. Such stress-related alterations (i.e., over-active SNS, under-active HPA axis), result in over-stimulation or disinhibition of inflammatory pathways and have typically been found in states of anxiety, depression, and stress (140, 141).

Through the pathway of inflammation, psychological and environmental effects can converge to negatively affect mental and physical health (142). In the context of climate change, psychological and environmental effects can negatively affect brain and periphery, not only in an additive, but also in a mutually potentiating way. For example, pollutant-induced weakening of the blood-brain barrier (89) would make it significantly easier for inflammatory cytokines—which can be the result of pollutants and/or psychological stress—to enter the brain. This can potentiate immune-to-brain signaling and thereby increase the development of further sickness-related psychological symptoms. A vicious cycle can develop, particularly in some vulnerable populations, with severe consequences for mental and physical health [see (142)].

### Cognitive Pathway

Climate and environmental stressors can impact mental health through their potential to influence key self-concepts and to induce non-adaptive cognitive defense mechanisms and coping strategies. These components of the cognitive pathway are detailed in the following sections.

#### Feelings of Identity

Humans can establish emotional bonds with their local environment. People-place bonds serve a functional purpose in the fulfillment of needs linked to solace, security, belonging, self-esteem, and identity (93). These people-place bonds and associated needs are threatened due to acute changes in the environment by natural disasters, or gradual changes that are either human-created or a consequence of climate change. This particularly affects individuals that live in close connection to the natural world, such as Indigenous populations, or those whose work and livelihoods largely depend on farmland (93). For instance, the Inuit of Northern Canada have a close connection to the land, and the changing environment has impacted their traditional way of living and induced profound changes in how they see and understand themselves. For example, in interviews they reported that “…it's who they are, it's what they've been grown up doing. And their parents have been doing it forever, so I mean they're kind of losing a sense of who they are” [(135), p. 20]; or that “…Inuit are people of the sea ice. If there is no more sea ice, how can we be people of the sea ice?” [Attutauniujut Nunami/Lament for the Land; www.lamentfortheland.ca; found in (16), p. 277]. This profound impact on the land, their way of living, and their perceived identity can elicit feelings of despair and even foster mental health issues. For example, research by Kumar and Tjepkema (143) found that the suicide rate was nine times higher for Inuit (72.3 suicides per 100,000 person-years at risk) than for non-Indigenous people (8.0 suicides per 100,000 person-years at risk). The Inuit, who have close connections to the land, are confronted by multiple stressors, among which is the climate and environmental crisis.

#### Cognitive Defense Mechanisms and Coping Strategies

Defense mechanisms and coping strategies, such as emotional-, cognitive-, or meaning-focused coping, positive reappraisal, or trust, can help individuals to deal with the unpleasant, undesirable, or unacceptable emotional states and thoughts elicited by the climate and environmental crisis. These mechanisms and strategies can also assist in creating feelings of constructive, active hope, as well as empowerment (39, 41). However, despite the known dangers of the climate and environmental crisis, resistance still exists against urgently needed mitigation activities. Explanations for this include the complex and abstract nature of climate change that is difficult to understand, visualize, and communicate; issues dealing with uncertainties; temporal discounting (i.e., placing a different value on short-term vs. long-term rewards); group dynamic processes (e.g., collective ignorance); or lack of personal relevance resulting in lack of urgency to act [e.g., (7)]. This is described in the Giddens Paradox: “It states that, since the dangers posed by global warming aren't tangible, immediate or visible in the course of day-to-day life—however awesome they may appear—many will sit on their hands and do nothing concrete about them. Yet waiting until they become visible and acute before being stirred to serious action will, by definition, be too late” [(144), p. 2].

Denial of the reality of climate change is a frequently observed cognitive response, particularly in conservative white males, conservative think tanks, or religious individuals (145, 146). This denial can be evoked for various reasons, including by mortality awareness, cognitive dissonance, protection of self- and group-identity and social norms, or system justification (145, 147, 148). The *Terror Management Theory* [TMT, (149)] offers one explanation for why the climate and environmental crisis can lead to strong denial responses in some individuals. The TMT states that the cognitive ability of self-awareness is coupled with the awareness of one's own mortality (130). Cognitive defense mechanisms can be engaged to deal with the thoughts of mortality elicited by the climate and environmental crisis: “…accessible death thoughts activate proximal defenses, for example, suppressing death-related thoughts *via* distraction or rationalization, or pushing the problem of death into the distant future and denying one's vulnerability. Cognitively inaccessible death thoughts activate distal defenses, for example, maintaining self-esteem and faith in one's cultural worldview” [(130), p. 3, 4].

Denial can also be a consequence of cognitive dissonance (147). Knowing that immediate global action is required calls for behavioral changes, which can be daunting and also hindered by various barriers, such as unwillingness to give up a comfortable lifestyle (41, 147). The conflict between being informed about the crisis and being unwilling or unable to take mitigating actions can create cognitive dissonance, which can be solved using denial mechanisms, such as rejection of blame or responsibility, or condemning the accuser (147). Furthermore, denial on a political level (by politicians) can provide false hope that there is no problem or threat, which can reinforce the belief that there is no reason for negative feelings [see (39)]. The complex and abstract nature of climate change, coupled with cognitive coping strategies can help explain the observed inaction toward the climate and environmental crisis.

### Behavioral Pathway

Climate and environmental stressors can also impact mental health *via* health-related behaviors. Health-damaging behaviors are often maladaptive responses to facilitate coping with (overwhelming) negative emotions and affective states (e.g., anger, fear), or unfavorable or harmful circumstances and environments [e.g., (150)]. An example of one health-damaging behavior is lack of physical activity, which has been associated with food insecurity (84), air pollution (86), and lower exposure to greenspace (151). In contrast, being surrounded by greenness has been shown to foster physical activity, which may positively impact weight and health [e.g., (152, 153)]. Surrounding greenspace has also been found to facilitate social cohesion (154). Both (outdoor) physical activities and positive social interactions and social cohesion have repeatedly been linked to beneficial impacts on mental health [e.g., (155–158)].

Another health-related behavior that is affected by climate change is sleep behavior (i.e., sleep time and quality), which can be impacted by heat (72, 159). Given the importance of a healthy sleep pattern for good mental health [e.g., (160)], this is an important pathway to consider in the understanding of how an ecological stressor (i.e., heat) can translate into poorer mental health.

Criminal activity and violence is a further set of behaviors that has previously been linked to changes and shifts in climate and weather and that has potential to affect physical and mental health. A study conducted in California that investigated the impact of drought on state-level rates of crime over an extended period of time found a meaningful increase in property (but not violent) crimes (161). A recently published study that analyzed survey data encompassing information from 80,000 women living in sub-Saharan Africa found that drought was linked to intimate partner violence, particularly in female adolescents and jobless women (162).

### Social Pathway

Given the impact of the climate and environmental crisis on multiple aspects of social life and socio-economic factors, combined with the fact that social determinants are meaningfully related to mental health (163), the social pathway is a key translator of climate and environmental stressors into mental health effects [e.g., (5)].

#### Destabilization of Social Relationships and Social Networks

Humankind is a social species and positive social interactions and social support are crucial for mental health, as they constitute important protective forces against the development of various mental health disorders, particularly PTSD (164). Climate and environmental stressors can destabilize social networks, interrupt social support chains, and reduce social cohesion. For example, food insecurity has been shown to lead to hopelessness and low levels of self-efficacy, which destabilized social relationships and increased the risk for depression (84). Furthermore, changes in urban social behavior have been observed as a result of air pollution, such as interacting less with neighbors (i.e., lower “social reciprocity”), which has been linked to depressive symptomatology (86). These studies illustrate the detrimental effect of the climate and environmental crisis on both social and mental health.

#### Impact on Socio-Economic Factors

Climate change, pollution, and destruction of the environment can impair an individuals' socio-economic status, and ultimately their mental health, by interrupting salary payments or education, loss of employment, financial constraints due to reduced agricultural production, crop failure, loss of livelihoods, or migration (131). For example, long-lasting heat waves and droughts have been shown to influence socio-economic hardship through the impact on work capacity, labor productivity, livelihoods, agricultural production, and food and water security (165). The complexity of this pathway is illustrated in a systematic review by Vins et al. (166) on the long-term mental health impact of drought. Drawing on data from 82 articles, the authors created a causal process diagram depicting the multiple indirect and interacting pathways between droughts and mental health. Drought was found to have multiple economic impacts, particularly for those dependent on weather conditions for their livelihood (e.g., farmers), which can result in financial hardship and endanger food security. This in turn can lead to stress, social isolation, interruption of education, worries about the future, denial, intimate partner violence, substance abuse, feelings of shame, depression, or suicide. This impact of drought (and the related economic effects) on mental health was shown to have a dose-response relationship, i.e., the more severe the drought, the worse the psychopathological consequences (166).

In connection to the economic effects, drought-related migration was further identified as having an important influence on mental health. The large-scale migration, resettlement, and displacement of people and populations are and will become necessary in response to the unfolding climate and environmental crisis, as a result of depressed economies and decreased environmental resources, such as food or water (166). This has the potential for a multitude of problematic social consequences, including disrupted social networks, loss of (land-bound) cultural traditions, acculturation stress, social turmoil, civil and international conflicts, and even wars (166–168). Regions of the world that struggle with corrupt political systems and social inequality are at higher risk of being affected by these developments (38). This research highlights the far-reaching socio-economic and related psychosocial health impacts of the climate and environmental crisis.

## Vulnerability and Adaptation

If the current rate of climate and environmental change is maintained, an overall increase in the adverse mental health impacts can be expected. However, individuals differ with respect to their vulnerability to ecological stressors, with the highest levels of vulnerability seen in those with pre-existing conditions (e.g., physical illnesses), higher exposure to ecological stressors, and reduced access to resources [e.g., (61, 113, 158)]. The following sections will describe risk and vulnerability factors, including vulnerable populations, as well as factors with the potential for increased adaptation in the face of climate and environmental stressors.

### Vulnerability

The climate and environmental crisis further increases existing inequalities by disproportionally affecting marginalized groups that are inherently vulnerable, for example, due to their development status (e.g., children), socio-economic disadvantage, or having mental health issues [e.g., (8)]. Such vulnerabilities do not act in isolation, but rather interact with each other. For instance, Indigenous people often live in geographical regions prone to extreme weather events, and typically have lower socio-economic resources and social power (7). Such economic, social, and gender inequalities are also risk factors for the development of psychopathology, and the added impact of climate and environmental stressors can lead to worse mental health (38, 132, 163).

#### Risk by Exposure

A differential impact on mental health is expected as a function of the magnitude of exposure to ecological stressors, with greater (peri- and post-) disaster exposure resulting in more adverse mental health effects (66, 67, 84, 169). For instance, high disaster exposure (as assessed by magnitude of disaster, property loss, witnessing someone dying, risk of own death, casualties, physical injuries, food/clothing insecurity, violence, or practical problems) has been shown to be a relevant risk factor for the development of (probable) PTSD (65, 70, 170, 171).

Exposure to the climate and environmental crisis is unequally distributed across the globe as a function of geographical regions that are more or less prone and sensitive to ecological stressors. More susceptible populations include those living in and dependent on rural landscapes (e.g., farming populations), in regions of dryland, in areas prone to floods, and in remote areas (48, 76). This vulnerability can be due to the geographical and social isolation, reduced infrastructure, lower levels of economic resources, or transport options (172). Developing countries, and particularly regions of the Global South, are also more vulnerable as they are more affected by water insecurity, steadily growing populations, increasing urbanization, and ever-present poverty (82, 120, 121, 132). Populations in urban regions are also exposed to ecological stressors, with a higher exposure to air pollution (85), as well as higher day- and night-time temperatures caused by a lack of (green) vegetation, dense architecture, and a higher temperature absorbance by urban surfaces (173). Urban residents are also exposed to more extreme weather events (e.g., thunderstorms) as urban areas intensify such weather events, combined with the fact that cities are often located close to water (i.e., rivers, sea), which increases the likelihood for floods (79). Additionally, urban life can be a stressful environment in itself, due to overpopulation, noise, crime, social stress, loneliness, and lower social support (85, 174). This differential exposure to climate and environmental stressors can render vulnerable individuals more susceptible to various physical and mental health conditions.

#### Indigenous People

Indigenous communities, particularly those living in remote areas, have a higher vulnerability to the climate and environmental crisis (166, 172). This includes individuals living in regions of the North (e.g., Inuit in Canada) who are (among other factors) affected by melting ice and alterations in flora and fauna; as well as Indigenous Australians who are affected by heat waves and bushfires (15, 172). The elevated vulnerability of Indigenous communities is due to their close dependence on and bond with nature for living, culture, and spirituality (48). Other vulnerability factors include susceptibility to poverty, a higher disease burden, low quality of infrastructure, and geographical remoteness that limits access to health-related (emergency) services (172).

#### Children and Adolescents

Minors are particularly vulnerable to the health impacts of the climate and environmental crisis: “According to the limited results from WHO, children under 5 years of age suffer the most from the consequences of climate change, with 88 percent of lost DALYs (disability-adjusted life years) attributable to climate change occurring in this age group in both developed and developing countries.” [(175), p. 33]. Ecological stressors can directly impact children and adolescents, for example, through natural disasters, *via* water-borne diseases, or air pollution. For instance, research on air pollution found that adolescents were vulnerable to the development of respiratory conditions in response to environmental toxins [e.g., (176)]; with other studies reporting evidence of cognitive dysfunction, damage in the prefrontal cortex area, or slower maturation [for a short review see (90)]. Such health impacts can affect the mental health of children and adolescents by interacting with existing mental health issues or by causing psychological sequelae (59, 177). Ecological stressors can also indirectly impact children and adolescents, such as through climate-related socio-economic hardship or migration (59); or through a lack of greenness exposure during early development, which has been linked to lower visuo-motor and language development [for a review see (151)].

Several explanations can be attributed to the increased vulnerability of minors. A major factor is that of the 2.2 billion children living worldwide, 1.9 billion are living in developing countries (178), many of which are disproportionally affected by ecological stressors. Another major risk factor is the biopsychological developmental phase. Children and adolescents have an underdeveloped physiology, limited cognitive and emotional abilities to deal with stressful circumstances, and are dependent on others [e.g., (74)]. As a consequence of these aspects of the biopsychological developmental phase, children and adolescents have a heightened vulnerability to stress. Stress experiences during childhood and/or adolescence can impact the brain structures and functions of the affected minors (179). This can impact their psychological development (e.g., emotion regulation issues, poorer cognitive abilities, learning and adjustment issues, or behavioral disorders) and increase their vulnerability to future stress experiences (74).

#### Older Adults

Older individuals are more vulnerable to climate and environmental stressors. For example, due to decreased mobility, functional limitations, increased frailty, and multimorbidity, older adults have a decreased likelihood to escape and survive (injuries from) disasters [e.g., (180)]. This can be seen in the age-bias of the fatalities from Hurricane Katrina: almost 50% of the deceased were older than 75 years, and up to 85% were older than 51 years (181). Older age was also found to be a meaningful predictor of higher posttraumatic stress after Hurricane Sandy (171). In addition, older individuals have a higher risk for the development of depression in response to climate change-related food insecurity (84).

Older adults are also more susceptible to extreme heat events (116). For example, research has linked warmer temperatures to higher suicide rates in older adults (182). Several explanations can account for the higher heat-related morbidity and mortality in older individuals: First, older individuals have a decreased physiological ability for thermoregulation, as well as the ability to sense, respond to, and recover from dehydration (116, 173). Second, higher age is linked to a higher prevalence for chronic health conditions, which can compound heat-related illnesses. Third, older adults have more heat-related risk factors, such as living alone, or smaller social networks (116, 173). Together, these aspects can increase older adults' vulnerability to (heat-related) ecological stressors.

#### Female Gender, i.e., Those Who Identify as Female

Women are considered to be more vulnerable to the effects of the climate and environmental crisis: “Climate change acts as a threat multiplier on already existing issues associated with gender inequality to increase the problem of marginalization faced by women” [(183), p. 2]. The reasons for this vulnerability vary depending on region and culture. In Europe, for instance, the higher vulnerability for women can be understood in relation to the increased likelihood of heat wave related fatalities in women [for a review see (115)]. In developing countries, the higher vulnerability can be linked to the structural disadvantages faced by many women, such as limited financial resources, and livelihoods that are largely dependent on the climate. The differential susceptibility of men and women can also be understood in relation to gender roles and discrimination in patriarchal societies, which can increase the risk exposure for women (183). For example, in many developing countries, fetching water is a physically straining task often carried out by children, girls, and women. This task is associated with many health risks, including falls, injuries, pain, fatigue, and a general lower well-being (82). It can be additionally dangerous due to a heightened risk for the sexual assault of females on the way to fetch water [for a review see (83)]. These factors can increase women's vulnerability to climate and environmental stressors.

#### Pre-existing Physical and Mental Health Conditions

Individuals at higher risk to be affected by the climate and environmental crisis (i.e., to present with psychopathology), include those with pre-existing health conditions and disabilities, those with higher stress levels or previous exposure to traumata, and those relying on (psychotropic) medication [e.g., (171)]. Individuals with (chronic) physical health conditions, such as cardiovascular or respiratory diseases or obesity, have a higher risk for heat-related morbidity and mortality, due to altered thermoregulation (116, 173). In addition, individuals with disorders that interfere with behavior, mobility, and/or awareness (e.g., dementia, Parkinson's disease) also have a higher risk for heat-related mortality (115). Furthermore, pre-existing mental health conditions can increase susceptibility to the impact of extreme weather events, such as an increased likelihood for PTSD (65). However, evidence on this is mixed. For example, in the study of estimated PTSD following Hurricane Katrina, no relationship was found between pre-existing psychopathology and PTSD trajectories (62). Certain medications can also affect thermoregulation or thirst-regulation (117, 118, 173). For instance, research on the 2003 heat wave in France revealed that anticholinergic, antipsychotic, and anxiolytic drugs were independently linked to hospitalization during the heat wave. This may be due to the detrimental impact of these medications on physiological thermoregulation (e.g., inhibited sweating) and behavioral thermoregulation (e.g., reduced water intake—drinking behavior) (184). This highlights the increased vulnerability to the impact of ecological stressors for individuals with pre-existing physical and mental health conditions.

#### Low Socio-Economic Status

Individuals with lower socio-economic resources, i.e., those with lower income, social status, or educational attainment, are more vulnerable to climate and environmental stressors (79, 165). For example, low-wage and extremely poor urban residents who have no or very limited isolation, as is the case in slums, are more strongly affected by heat or heavy rain (79). Low-income and education are also linked to a higher heat-related mortality risk [for a review see (116)]. Furthermore, increasing temperatures can cause heat stress and decrease work productivity and increase the likelihood for injury, which disproportionally affects countries with warmer climates. For example, a national cohort study in Thailand with almost 60,000 workers, showed that one-fifth of the workers reported occupation-related heat stress and that this was meaningfully linked to a higher risk of injuries (109). In addition, individuals with disadvantaged backgrounds often live in areas with reduced access to green spaces and lower tree canopy cover, with decreased exposure to the health-promoting influence of greenness [e.g., (157)]. These multi-faceted aspects of low socio-economic status can increase vulnerability to the impact of the climate and environmental crisis.

### Adaptation

There is a repeatedly observed, yet insufficiently understood heterogeneity in how individuals respond and adapt to adversity. While it is essential to consider risk and vulnerability factors in explaining this heterogeneity, protective factors are equally as important in understanding interindividual differences in adapting to the climate and environmental crisis and the impact on mental health. This positive adaptation and potential protective factors will be outlined in the following sections.

#### Resilience

Resilience can be broadly defined as positive adaptation in the face or aftermath of stress and adversity [e.g., (185)]. Resilience is not an uncommon phenomenon and with respect to acute disasters, it has been shown that the majority of individuals can adapt well (22). For example, longitudinal research by Lowe and Rhodes (169) found that more than 60% of participants showed a resilient trajectory after Hurricane Katrina. Another longitudinal study with Mexican individuals affected by a large-scale flood found that 35% of participants showed a resistant trajectory (stable, mild symptomatology), and 31% showed a resilient trajectory (initial severe symptomology that declined to a moderate symptom level) (186). Evidence of resilience may also be seen for gradual changes to the natural environment, such as that experienced by the Inuit in Canada: “…there is nothing else (we can do), we can't dwell on it. Then we would be all suicidal. You just have to do the best you can with what change is coming” [(15), p. 265]. Such resilience varies as a function of internal and external factors [e.g., (187)].

Internal psychological factors that have been linked to higher adaptability to the climate and environmental crisis include self-efficacy (188), sense of optimism, being informed, social support (63, 169), and increased (access to) pre-disaster resources (189). In climate scientists, a source of resilience can be seen in the sense of community, i.e., the feeling that one is not alone in combating the climate change but that one is working in a community with a shared goal; this can assist in increasing the perception of being supported and validated (190).

External resilience factors include the general resilience of communities, as well as specific aspects, such as how prepared the health-care sector is to handle disasters and environmental risks (68). An important external factor is vicinity or exposure to surrounding greenness and outdoor blue spaces, such as lakes (105, 191, 192). A large and steadily increasing number of studies demonstrate the health-promoting effects of being in and interacting with nature [e.g., (153, 154)], of viewing natural landscapes [e.g., (193)], or even listing to natural sounds [e.g., (194)]. Increased exposure to environmental greenness has been linked to lower psychological distress, depression and anxiety disorders, and schizophrenia incidence (105, 157, 195); as well as to slower cognitive decline in middle-aged individuals (191); and improved self-reported mental health in urban settings [see (196)]. Greenness is also important for healthy development in children, with a recent systematic review identifying an association between exposure to greenness and decreased emotional and behavioral difficulties, particularly inattention and hyperactivity (151). Furthermore, surrounding greenness can decrease the ambient and surface temperature in cities, and thus counteract the negative health effects of urban heat (105). Greenspace has also been found to physically reduce exposure to (traffic) noise and to buffer the noise-induced psychological stress response (154).

The positive impact of nature on emotional well-being has also been observed for Indigenous people who have close bonds with the land. For example, research with Inuit in Canada found that if they were unable to be outside and interact with nature, participants reported feelings of craving and being caged inside like an animal, which was stressful and depressing (15, 135). In contrast, being closely connected to nature elicited positive emotions and increased the feeling of being healthy. For instance, one participant reported that “…the air and the land takes a lot of your feelings away and replaces the negative energy with the positive energy, nature …” [(15), p. 262]. The above research suggests that certain (internal and external) factors can foster positive adaptation (i.e., resilience) to climate and environmental stressors.

#### Adversity-Related Positive Development

Some individuals may not only adapt well in the face of adversity, but may also experience positive interpersonal development [e.g., (197)]. An example of this can be seen in the research on Hurricane Katrina (198). Results not only showed a significantly lower conditional prevalence of suicidality post-hurricane (suicidal ideation: 0.7%; suicidal plans: 0.4%), compared pre-hurricane (suicidal ideation: 8.4%; suicidal plans: 3.6%); but also revealed that suicidality was linked to aspects of posttraumatic growth, such as becoming aware of one's inner strength or developing faith in the capability to rebuild one's life (198). Another example is provided by a recent qualitative study that involved interviews on lived experiences by individuals who were personally affected by severe wildfires in Canada: It was found that besides negative feelings of distress, anxiety and uncertainty by their evacuation and isolation, positive aspects were reported, too, such as the creation of opportunities for supporting and caring for one another (61).

Taken together, the above research indicates that while it is important to consider risk and vulnerability to ecological stressors, protective factors can also facilitate positive adaptation and even development in response to the climate and environmental crisis.

## Methodological Considerations and Future Directions

In the exploration of the literature on climate change and mental health, methodological limitations must also be considered to inform directions for future research.

### Methodological Limitations

Given the vast complexities of the two factors involved (i.e., anthropogenic climate change and mental health), attributing a causal link is difficult and poses multiple methodological challenges (22). While an increasing number of studies have examined the adverse mental health effects of the climate and environmental crisis, much of the available data is based on (retrospective) cross-sectional research, or qualitative, case-study and ethnographic designs. These studies lack information on trajectories and long-term effects and prohibit the establishment of causal connections (83). Baseline data (i.e., pre-disaster mental functioning) is often lacking [valuable exceptions are (63, 64, 66)]. This hinders statements about change in psychopathology in response to the event, as well as the examination of predictors related to interindividual variability in adaptation (199). The mental health impact of acute, isolated events (e.g., hurricanes) can be more easily assessed, such as by examining the change in mental functioning prior- (if available) to post-disaster. However, the attribution of (all) these extreme weather events to climate change is problematic; as while their increased frequency or power may vary as a function of the changing climate, their occurrence may not (8, 131). Attributing the mental health impact of sub-acute and chronic phenomena is also challenging, as they often do not have a defined beginning or ending and their impact is often experienced indirectly due to the subtle, gradually-evolving, and accumulating nature of these types of events (8).

With regard to mental health, the majority of studies assessed mental health disorders with self-report screening instruments, which are prone to subjective bias, are less precise than structured clinical interviews, and limit conclusions to probable/estimated mental health disorders. In addition, many studies did not include control groups, or neglected to control for relevant confounding influences (67, 85). It should also be taken into consideration that mental health disorders can vary as a function of norms and cultural contexts and their etiology depends on multiple biopsychosocial factors (11). Furthermore, the assessment of adverse mental health effects in post-disaster populations is often affected by sample biases. For example, those who are most marginalized, those affected most by (post)disaster-related stressors, and those with the highest psychopathological burden are less likely to participate in studies, which bears the potential for underestimation of mental health effects (62, 170). Mental health disorders are also stigmatized in many cultures, which can impact disclosure and assessment (200).

### Recommendations for Future Studies

In addition to addressing these methodological limitations identified in previous studies, some specific recommendations should also be considered for future research. A stronger focus should be placed on underlying processes, particularly emotional, cognitive, and behavioral processes that have previously been neglected. Future studies should also examine potential positive changes that can occur as a result of the climate and environmental crisis, such as post-disaster psychological growth; increased sense of meaning, social engagement, and social cohesion compassion; or increased engagement with mitigation activities (8, 38, 198).

Going forward, mixed-methods approaches are recommended, as they combine qualitative and quantitative methods that are suitable to assess complex relationships, such as the adverse mental health impact of sub-acute weather events (166). Mixed-methods also allow for a person-centered approach and an in-depth analyses and understanding of rich data, as well as the quantification of the mental health burden (67). There is also a need to move beyond the current focus of conventional epidemiology, which “…focuses too much on the individual, on direct or proximate causes, on the past and present, and on current states of being; and too little on whole populations, indirect and distal influences, the life course and the future, and the dynamics of health across contexts” [(131), p. 283]. Future research could apply a systems approach (i.e., combining various perspectives from multiple academic disciplines and perspectives) to better foster an understanding of the intertwined factors and processes underpinning climate change and mental health [see (131)]. With regard to data assessment analysis methods, large-scale, longitudinal, population health studies and experimental epidemiological studies could best fit this approach, using network- and data-driven analyses, as well as agent-based and dynamic models (131).

## Conclusion

Climate change is not only one of this century's major challenges, but is probably one of the most important challenges in the history of humankind. Given the dependence of humans on a healthy environment (124), the mitigation of climate change and protection of the natural environment must become a top priority. A change toward the responsible management and protection of the natural world and a more sustainable lifestyle may ultimately foster better mental health [e.g., (5)]: “The good news is that such transformative change, with social and economic justice for all, promises far greater human well-being than does business as usual” [(3), p. 11].

A growing number of studies are examining the mental health impact of the anthropogenic climate change, pollution, and destruction of the natural environment. However, the climate and environmental crisis, as well as mental health, are highly complex topics; and as this field of study is still in its early days, the underlying processes remain insufficiently understood. This synergy of literature has aimed to provide a current summary of existing research on the mental health impact of the climate and environmental crisis from the perspective of clinical psychology. Given the urgency of the current situation, it is of crucial importance that future research examines this neglected relationship in light of the identified processes and pathways, including the consideration of potential vulnerability and protective factors. We herewith call for increased investigations into this topic by clinical psychologists, within the context of this rapidly growing research area of Clinical Ecopsychology.

## Author Contributions

MT: definition, conceptualization, writing of original draft, and project administration. NR and SR: writing parts of the manuscripts, reviewing, and editing. All authors contributed to and have approved the final manuscript.

## Conflict of Interest

The authors declare that the research was conducted in the absence of any commercial or financial relationships that could be construed as a potential conflict of interest.
